# Epithelium-specific Ets transcription factor-1 acts as a negative regulator of cyclooxygenase-2 in human rheumatoid arthritis synovial fibroblasts

**DOI:** 10.1186/s13578-016-0105-7

**Published:** 2016-06-16

**Authors:** Chan-Mi Lee, Sahil Gupta, Jiafeng Wang, Elizabeth M. Johnson, Leslie J. Crofford, John C. Marshall, Mohit Kapoor, Jim Hu

**Affiliations:** SickKids Research Institute, Program in Physiology and Experimental Medicine, The Hospital for Sick Children, Peter Gilgan Centre for Research and Learning, 9th floor, 686 Bay Street, Toronto, ON M5G 0A4 Canada; Laboratory Medicine and Pathobiology, University of Toronto, 1 King’s College Circle, Toronto, ON M5S 1A8 Canada; The Keenan Research Centre, Li Ka Shing Knowledge Institute, St. Michael’s Hospital, 209 Victoria Street, Toronto, ON M5B 1T8 Canada; Institute of Medical Science, Faculty of Medicine, University of Toronto, 1 King’s College Circle, Toronto, ON M5S 1A8 Canada; Department of Surgery, St. Michael’s Hospital, University of Toronto, 30 Bond Street, Toronto, ON M5B 1W8 Canada; Department of Anesthesiology and Intensive Care, The Second Military Medical University, Changhai Hospital, Shanghai, 200433 China; Department of Medicine, Division of Rheumatology and Immunology, School of Medicine, Vanderbilt University, 1161 21st Ave S, MCN T-3113, Nashville, TN 37232 USA; Division of Genetics and Development, Toronto Western Research Institute, Toronto Western Hospital, University Health Network (UHN), 60 Leonard Avenue, Toronto, ON M5T 2S8 Canada

**Keywords:** Rheumatoid arthritis, ESE-1, COX-2, Inflammation, Synovial fibroblasts, Helper-dependent adenovirus, Prostaglandins

## Abstract

**Background:**

Rheumatoid arthritis (RA) is characterized by excessive synovial inflammation. Cyclooxygenase-2 (COX-2) is an enzyme that catalyzes the conversion of arachidonic acid (AA) into prostaglandins. Epithelium-specific Ets transcription factor-1 (ESE-1) was previously demonstrated to upregulate COX-2 in co-operation with nuclear factor kappa B (NFκB) in macrophages and chondrocytes. However, the role of ESE-1 in RA pathology has remained unclear. In this study, we aimed to elucidate the relationship between ESE-1 and COX-2 in RA synovial fibroblasts (RASFs) using a HD-Ad-mediated knockdown approach.

**Results:**

ESE-1 and COX-2 were induced by IL-1β in RASFs that corresponded with an increase in PGE_2_. Endogenous levels of ESE-1 and COX-2 in human RASFs were analyzed by RT-qPCR and Western blot, and PGE_2_ was quantified using competitive ELISA. Interestingly, knockdown of ESE-1 using helper-dependent adenovirus (HD-Ad) led to a significant upregulation of COX-2 at a later phase of IL-1β stimulation. Examination of ESE-1 intracellular localization by nuclear fractionation revealed that ESE-1 was localized in the nucleus, occupying disparate cellular compartments to NFκB when COX-2 was increased. To confirm the ESE-1-COX-2 relationship in other cellular systems, COX-2 was also measured in SW982 synovial sarcoma cell line and ESE-1 knockout (KO) murine macrophages. Similarly, knockdown of ESE-1 transcriptionally upregulated COX-2 in SW982 and ESE-1 KO murine macrophages, suggesting that ESE-1 may be involved in the resolution of inflammation.

**Conclusion:**

ESE-1 acts as a negative regulator of COX-2 in human RASFs and its effect on COX-2 is NFκB-independent.

**Electronic supplementary material:**

The online version of this article (doi:10.1186/s13578-016-0105-7) contains supplementary material, which is available to authorized users.

## Background

Rheumatoid arthritis (RA) is a systemic autoimmune disease characterized by the progressive destruction of the joints due to excessive inflammation in the synovium, which can lead to deformities and loss of joint function in severe cases. Inflammation in the RA synovial tissue is perpetrated by the production of inflammatory cytokines and secreted mediators from infiltrating immune cells and activated synovial fibroblasts [1]. Prostaglandins (PGs) are key mediators responsible for RA symptoms of pain and swelling [2]. Synthesis of PG requires conversion of arachidonic acid released from cell membranes to prostaglandin H_2_ (PGH_2_), the critical step of which is catalyzed by cyclooxygenase-2 (COX-2), also known as the PGH_2_ synthase. PGH_2_ is further metabolized to bioactive forms such as PGE_2_, prostacyclin, prostaglandin D_2_, and prostaglandin F_2α_, by their respective synthases in different cell types [2, 3]. COX-2 is highly expressed in the RA synovial lining due to the persistent presence of proinflammatory cytokines such as IL-1β, TNF-α, and IL-6, and is a key biosynthetic enzyme regulating PG production in the synovium [4, 5]. PGE_2_ is the major PG that is generated by chondrocytes and synovial fibroblasts [6], and clinical responses to non-steroidal anti-inflammatory drugs (NSAIDs) have been shown to correlate with reduced levels of PGE_2_ in the synovial fluid [7, 8]. COX-2 inhibitors such as celecoxib effectively control arthritis symptoms [8].

COX-2 gene activation is complex and employs numerous regulatory factors specific to different stimuli, as exemplified by the COX-2 promoter which contains two NFκB motifs, two activator protein 1 (AP-1) sites and two cAMP-response elements (CREs) among others [9]. Several Ets factors have also been shown to regulate COX-2 expression, including Ets-1 [10], Pea3 [11] and PU.1 [12], and Elk1 [13] in different tissue contexts. Ets family of transcription factors are characterized by the highly conserved E26 transformation-specific (Ets) DNA binding domain, which recognizes GGAA/T core consensus sequence within the promoter and enhancer regions of target genes [14]. Unlike most Ets factors which are expressed in hematopoietic cells, however, a subgroup of Ets proteins called epithelium-specific Ets factors (ESE’s) has epithelium-restricted expression pattern under basal conditions. Interestingly, ESE-1, the prototype of ESE subfamily, is highly sensitive to inflammatory stimulation [15], where it was found to be expressed in the human RA synovial tissue [16]. It was also transcriptionally upregulated by proinflammatory stimuli such as IL-1β, TNF-α, or LPS in the resident cell types including synovial fibroblasts, chondrocytes, osteoblasts, and macrophages, typically displaying a peak expression between 2–6 h and dissipation by 24 h in most cells [16]. ESE-1, or Elf3 in mice, was similarly found to transactivate COX-2 promoter in murine macrophages and human chondrocytes in cooperation with NFκB [17], suggesting its critical role in RA pathogenesis. However, initial analyses had revealed ESE-1 to be predominantly expressed in the cytoplasm of the cells [16], leaving discrepancies in how it might function as a transcription factor in situ. Additionally, the prolonged expression of ESE-1 mRNA in RASFs beyond 24 h of IL-1β stimulation unlike in other cell types has left the relationship between ESE-1 and COX-2 in RASFs elusive. As RASFs and synovial macrophages are prominent cell types present in the terminal layer of the hyperplastic synovial tissue which secrete inflammatory cytokines and matrix-degrading enzymes [18, 19], elucidation of the role of ESE-1 in COX-2 regulation is important to gain better understanding of the molecular events that occur in RA synovial tissues.

Previous studies primarily focused on investigating functional significance of ESE-1 by ESE-1 overexpression, where ESE-1 cDNA was transfected into cell lines along with luciferase constructs to investigate the transactivation of ESE-1 on its target genes. However, ectopic gene expression can lead to supraphysiological levels of the gene of interest, as well as cell toxicity from the transfection procedure itself. Also, overexpression by transfection may not accurately reflect the temporal behaviour of a protein, and may thus lead to artificial interaction or co-localization of proteins that do not normally co-exist under physiological conditions. ESE-1 overexpression could also have accompanied co-induction of its other target genes, giving rise to confounding results. Therefore, we sought to elucidate the relationship between ESE-1 and COX-2 in human RASFs using a knockdown approach with helper-dependent adenoviral (HD-Ad) vector, which has all of the viral genes removed to render it much less immunogenic than conventional adenoviruses [20, 21], and in Elf3 knockout mouse bone marrow-derived macrophages (BMDMs) to avoid side effects from transfection- or transduction-mediated gene manipulation. In this study, we show for the first time that ESE-1 negatively regulates COX-2 in human RASFs.

## Methods

### Reagents

DMEM, RPMI, fetal bovine serum (FBS) and l-glutamine were purchased from Gibco Life Technologies Ltd., Burlington, Ontario, Canada. Penicillin/streptomycin, phosphate buffered saline (PBS) were from Wisent, St. Bruno, Quebec, Canada. Human recombinant IL-1β was product of R&D Systems (Minneapolis, MN, USA), and LPS endotoxin (*Escherichia coli,* serotype O128:B12) and DEAE-Dextran hydrochloride of Sigma (Oakville, Ontario, Canada), while murine IL-4 was from Peprotech, Quebec, Canada. Antibodies used in this study were: COX-2 rabbit polyclonal antibody from Thermo Fisher Scientific (Burlington, Ontario, Canada), and COX-2 (C-20) goat polyclonal, NFκB p65 (C-20), p50 (H-119) and Lamin A (H-102) rabbit polyclonal antibodies from Santa Cruz (Dallas, TX, USA). ESE-1 rabbit monoclonal antibody was produced in our laboratory in collaboration with Epitomics, Burlingame, CA, USA [22]. Hsp90 rabbit polyclonal and β-actin mouse monoclonal antibodies were purchased from Cell Signaling Technology (Whitby, Ontario, Canada).

### Preparation of RASFs

Synovial tissues were obtained at the time of joint replacement surgery from patients with RA who fulfilled the revised American Rheumatism Association criteria for this disease [7]. Experiments were carried out according to a protocol that was approved by the Institutional Review Board in Vanderbilt University, Nashville, TN, and patient informed consent was obtained. RASF were prepared as previously described [9]. Briefly, minced synovial tissues were digested overnight with 1 mg/ml collagenase (Type I, Sigma, St. Louis, MO, USA) in DMEM in a humidified 5 % CO2 incubator at 37 °C and the isolated cells were cultured in 175 cm^2^ culture flasks in DMEM supplemented with 20 % FBS, l-glutamine (2 mM), penicillin (100 units/ml) and streptomycin (100 μg/ml). At greater than 95 % confluency, the adherent RSF were passaged by digestion with 0.05 % trypsin/EDTA and used for cell culture experiments.

### Cell culture

Human synovial sarcoma and lung adenocarcinoma cell lines SW982 and A549 were obtained from American Type Culture Collection, Rockville, MD, and were cultured in DMEM supplemented with 10 % FBS and 100 IU/mL penicillin, and 100 μg/mL streptomycin in 5 % CO_2_ at 37 °C. Cells were starved in serum-deprived medium containing 0.5 % FBS, in which transduction and cytokine stimulation were also performed.

### Infection of cells with helper-dependent adenovirus (HD-Ad)

*ESE*-*1* gene was knocked down in human synovial fibroblasts and SW982 cells using shRNA helper-dependent adenoviral vector expressing two shRNAs prepared as previously described [23, 24], with added modifications from [25]. Briefly, cells were seeded at 100,000 cells per well in growth medium on 6-well plates a day prior to transduction, and 5000 virus DNA particles per cell equivalent of 100 MOI were complexed with 520.5 ng DEAE-Dextran by incubation for 30 min at room temperature in 0.5 % FBS DMEM. The DEAE-virus mixture was added to cells by replacing the growth medium. C4HSU empty vector was used as control. The cells were incubated for 2 h in a 5 % CO_2_ at 37 °C, after which 20 % FBS DMEM was added to achieve a final concentration of 10 % FBS. The cells were then incubated for additional 48 h, and the medium was removed and replaced with 0.5 % FBS DMEM for 24 h starvation before being stimulated with 10 ng/mL IL-1β.

### RNA isolation and real-time quantitative PCR (RT-qPCR)

Total RNA was isolated using GE Illustra RNAspin Mini Kit (GE Healthcare Life Sciences, Baie-D’Urfe, Quebec) as per manufacturer’s instructions. For real-time quantitative PCR, after spectrophotometry quantification, 1 µg of RNA was reversed transcribed in a final volume of 20 μL using Superscript VILO Mastermix with Superscript III (Invitrogen, Carlsbad, CA) and the resulting cDNA template (10 ng) was used for qPCR reaction using Power SYBR Green PCR Master Mix from Life Technologies (Burlington, Ontario, Canada). ViiA™7 Real-Time 384-well PCR System from Life Technologies was used for the amplification and analysis. For relative ΔΔCt quantification, qPCR signals were normalized using GAPDH and fold changes were calculated according to Livak and Schmittgen [26]. The primer sequences used for human and mouse samples are provided in Additional file 1: Table S1.

### Cytoplasmic nuclear fractionation and Western blot

Nuclear and cytoplasmic extracts were prepared from human RASFs by nuclear/cytoplasmic separation as previously described [27]. In summary, cells grown in 10 cm dishes were washed twice with ice-cold PBS, and pelleted cells were resuspended in 900 µL of hypotonic buffer containing 0.1 % NP-40 in PBS containing protease inhibitors (Roche; Mississauga, Ontario, Canada) and triturated five times, after which they were immediately centrifuged at 500×*g* at 4 °C. The supernatant was collected and designated as the cytoplasmic extract, while the pellet was washed once with 1 mL of 0.1 % NP-40 PBS buffer, re-centrifuged, and lysed in 180 µL 6 × SDS sample buffer [2 % (w/v) SDS, 58.3 mM Tris–HCl (pH 6.8), 6 % (v/v) glycerol, 5 % (v/v) 2-β-mercaptoethanol, 0.02 % (w/v) bromophenol blue] and was designated as the nuclear extract. The nuclear extract was sonicated at Level 2 on Misonix 3000 sonicator for 5–10 s. Lysates were separated by electrophoresis on 10 % SDS-PAGE gel, and transferred to nitrocellulose membrane (Amersham; GE Healthcare, Mississauga, Ontario, Canada). Membranes were blocked with 5 % (w/v) nonfat milk in TBST (50 mM Tris–Cl pH 7.5, 150 mM NaCl, 0.05 % Tween-20) for 1 h at room temperature and probed for ESE-1 (1:3000), COX-2 (1:500), Hsp90 (1:1000), β-actin (1:4000), or Lamin A (1:500) overnight at 4 °C. Protein signals were detected with HRP-conjugated secondary antibodies at a dilution of 1:4000 using ECL Western blotting detection system (Amersham Pharmacia Biotech, Baie-D’Urfe, Quebec, Canada).

### Prostaglandin E_2_ (PGE_2_) quantification

PGE_2_ was quantified using a competitive binding ELISA kit (R&D Systems) according to the manufacturer’s protocol. Cell medium was centrifuged to remove particulates and the supernatant was diluted threefold before the assay. The plate was read with plate reader VersaMaxPLUS ROM v1.21 with SoftMax Pro v5.3b12 software at the absorbance of 450 nm with wavelength correction at 540 nm to correct for the optical imperfections in the plate. The concentration of PGE2 was calculated against a standard curve ranging from 0 to 2500 pg/mL.

### ESE-1/Elf3 knockout mice and bone marrow-derived macrophage culture

Elf3-/- mice on a C57BL/6 background were housed in pathogen-free condition at Toronto Centre for Phenogenomics (TCP), Toronto, Canada, and all procedures were approved by the Toronto Centre for Phenogenomics Animal Care Committee (Animal Use Protocol #0062). Bone marrow was flushed from femur and tibia of Elf3−/− mice and wild-type littermates into single cell suspension and cultured in 20 % L-929 conditioned media containing for 7 days as previously described [28]. Wild-type littermates were used as controls. The purity of bone marrow-derived macrophages was measured by flow cytometry with CD11b and F4/80 following methods from [10]. 4 × 10^5^ of mature BMDMs were subjected to 100 ng/mL LPS or 10 ng/mL IL-4 for 18 h to drive M1 and M2 polarization, respectively.

### Statistical analysis

Statistical analysis was performed by two-tailed Student’s *t* test with Welch’s corrections for unequal variances where appropriate, or by one-way paired ANOVA for multiple comparisons with Tukey’s post-test or Bonferroni’s post-test for selected pairs using GraphPad Prism 5.03 (GraphPad Software, Inc., La Jolla, CA, USA). P values of less than 0.05 were considered statistically significant.

## Results

### ESE-1 and COX-2 are induced by IL-1β in human RASFs

**Fig. 1:**
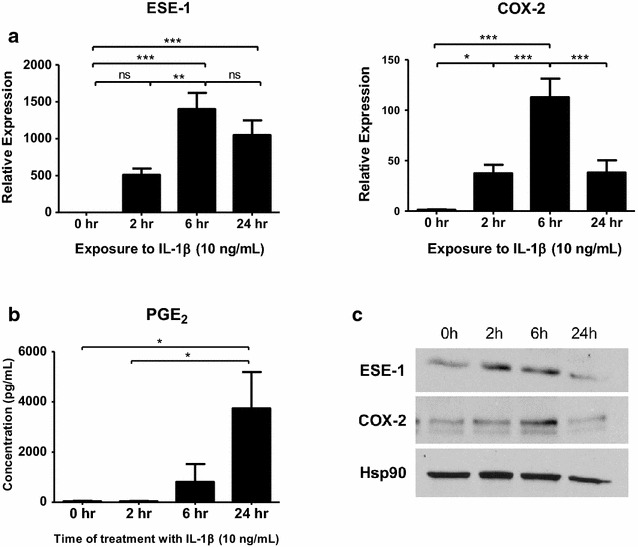
ESE-1 and COX-2 are induced by IL-1β in human RASFs. **a** Changes in ESE-1 and COX-2 transcriptional levels in human patient RASFs (n = 5) during IL-1β (10 ng/mL) stimulation by quantitative RT-PCR normalized to GAPDH. **b** Quantification of prostaglandin E2 (PGE_2_) in the culture media of human RASFs (n = 6) stimulated with 10 ng/mL of IL-1β for the designated time points. **c** Representative Western blot from four independent patients showing changes in ESE-1 and COX-2 at protein level, using Hsp90 as a loading control. *Bars* show mean ± SEM, *P < 0.05, **P < 0.01, ***P < 0.001

ESE-1 was previously shown to be rapidly upregulated by proinflammatory stimuli in human RASFs and maintained up to 24 h [15, 16]. To investigate its relationship to COX-2 expression, we stimulated primary RASFs with 10 ng/mL human IL-1β and quantified the amount of ESE-1 and COX-2 mRNA by RT-qPCR (Fig. 1a). We observed that ESE-1 mRNA expression peaked around 6 h and was reduced slightly at 24 h. Transcriptional levels of COX-2, on the other hand, showed gradual increase until 6 h and downregulation at 24 h post IL-1β stimulation. Protein levels of ESE-1 varied among RA patients, though a representative is shown in Fig. 1c, indicating heterogeneity of the patient population and potentially the presence of other factors which may modulate ESE-1 expression (Additional file 2: Figure S1A). COX-2 protein, however, accumulated over time in most patients as previously shown [29] (Additional file 2: Figure S2B), which correlated with increase in PGE_2_ concentration (Fig. 1b).

### RASFs can be effectively transduced by helper-dependent adenovirus (HD-Ad)

**Fig. 2:**
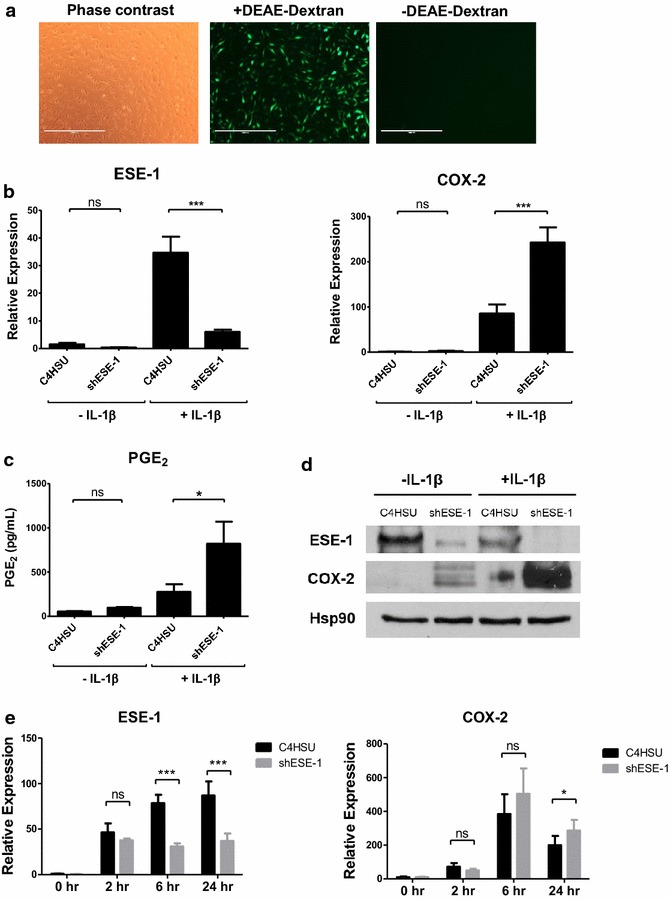
Knockdown of ESE-1 leads to increased COX-2 and PGE_2_ production in RASF. **a** Representative picture of human RASFs successfully transduced with helper-dependent adenovirus containing EGFP construct (HD-Ad-EGFP) with and without DEAE-Dextran. **b** Knockdown of ESE-1 leads to the transcriptional upregulation of COX-2 following 24 h of IL-1β stimulation (n = 6). The cells were incubated in the presence of HD-Ad and DEAE-Dextran complex for 48 h and starved in 0.5 % FBS DMEM for 24 h prior to incubation with IL-1 β.* Scale bar* shows 100 μm. **c** Transcriptional upregulation of COX-2 is accompanied by increased production of PGE_2_ (n = 6). **d** Representative *Western blot* showing changes in ESE-1 and COX-2 proteins in RASFs transduced with control (C4HSU) or shESE-1 HD-Ad. *Bars* show the mean ± SEM, *P < 0.05, **P < 0.01, ***P < 0.001, by one-way ANOVA Bonferroni’s post-test. (E) Time point analysis of ESE-1 and COX-2 transcriptional levels (n = 5) during IL-1β stimulation after C4HSU or shESE-1 HD-Ad viral transduction. *ns* not significant, *P < 0.05, ***P < 0.001

Helper-dependent adenovirus (HD-Ad) provide an attractive alternative means of gene delivery to non-viral vectors or other virus types, by its high carrying capacity of 37 kb and low immunogenicity from having all its viral coding sequences removed [20, 21]. HD-Ads have been successfully produced and used in our laboratory as a potential tool for cystic fibrosis (CF) gene therapy [30], as well as a research tool to knockdown ESE-1 in a number of studies [23, 24]. However, similar to other adenoviruses, HD-Ad requires specific receptors to mediate viral attachment and gene transfer, notably the coxsackie virus and adenovirus receptor (CAR), which the fibroblasts are known to be lacking [31]. As expected, transduction with virus alone in human RASFs proved ineffective irrespective of viral dose (Fig. 2a), despite yielding close to 100 % transduction in A549 lung adenocarcinoma cell line (data not shown). Non-covalent complexing of recombinant adenovirus with cationic molecules, however, has been demonstrated to significantly increase viral attachment and thus the efficiency of gene transfer by neutralizing the net negative surface charge on virus particles and the cell membrane [25, 32]. In an attempt to optimize viral infection in human RASFs, therefore, we complexed HD-Ad with DEAE-Dextran, which resulted in 100 % cells being transduced at 100 MOI, with the expression lasting for more than 96 h. We were able to achieve up to 90 % knockdown of ESE-1 with HD-Ad carrying shESE-1 construct using this infection protocol (Fig. 2b). Although it was inevitable that the virus led to some degree of inflammation and ESE-1 activation, by 72 h from the initial exposure to virus particles, the background PGE_2_ and COX-2 expression were comparable to basal levels prior to IL-1β stimulation. There was also minimal cell toxicity, if any, conferring a significant advantage over transfection. This indicates that with optimization with charge-neutralizing polymers such as DEAE-Dextran, HD-Ad can be an effective gene delivery tool for hard-to-transduce cell types such as immune cells and fibroblasts, and to study immune-responsive or cell survival genes that can be affected by transfection.

### Knockdown of ESE-1 leads to upregulation of COX-2 and increased PGE_2_ production

In all patient RASF studied, knockdown of ESE-1 led to a significant upregulation of COX-2 at both RNA (Fig. 2b) and protein (Fig. 2d) levels. This correlated with increased concentration of PGE_2_ in the cell media (Fig. 2c), indicating functional significance of ESE-1 on COX-2 activity. There was also a recognizable heterogeneity in different patient RASFs resulting in different basal expression of ESE-1 following HD-Ad transduction (Additional file 2: Figure 1c), but all showed similar trends of upregulated COX-2 following ESE-1 knockdown. Interestingly, however, ESE-1 knockdown had no effect on COX-2 basal level of mRNA expression or early phase of induction, and the effect of ESE-1 knockdown on COX was only visible at 24-hour time point (Fig. 2e). Similarly, while adenovirus binding to cultured synoviocytes is known to trigger COX-2 expression through the MAPK pathway, this subsides by 24 h [33] and in our study, transduced cells were stimulated with IL-1β 72 h after initial exposure to the virus when both COX-2 and PGE_2_ were comparable to the basal levels, thus minimizing confounding results from the inflammatory reaction to the viral vector. Still, C4HSU empty vector control virus was used in all experiments to account for the basal inflammatory response to the viral vector itself. Additionally, knockdown of ESE-1 had no effect on metalloprotease activity in RASFs, as shown by insignificant changes in MMP-1 or -13 mRNA expression (Additional file 2: Figure S2), indicating that ESE-1 may be a specific effector for resolving inflammatory responses.

### ESE-1 is localized in the nucleus

**Fig. 3:**
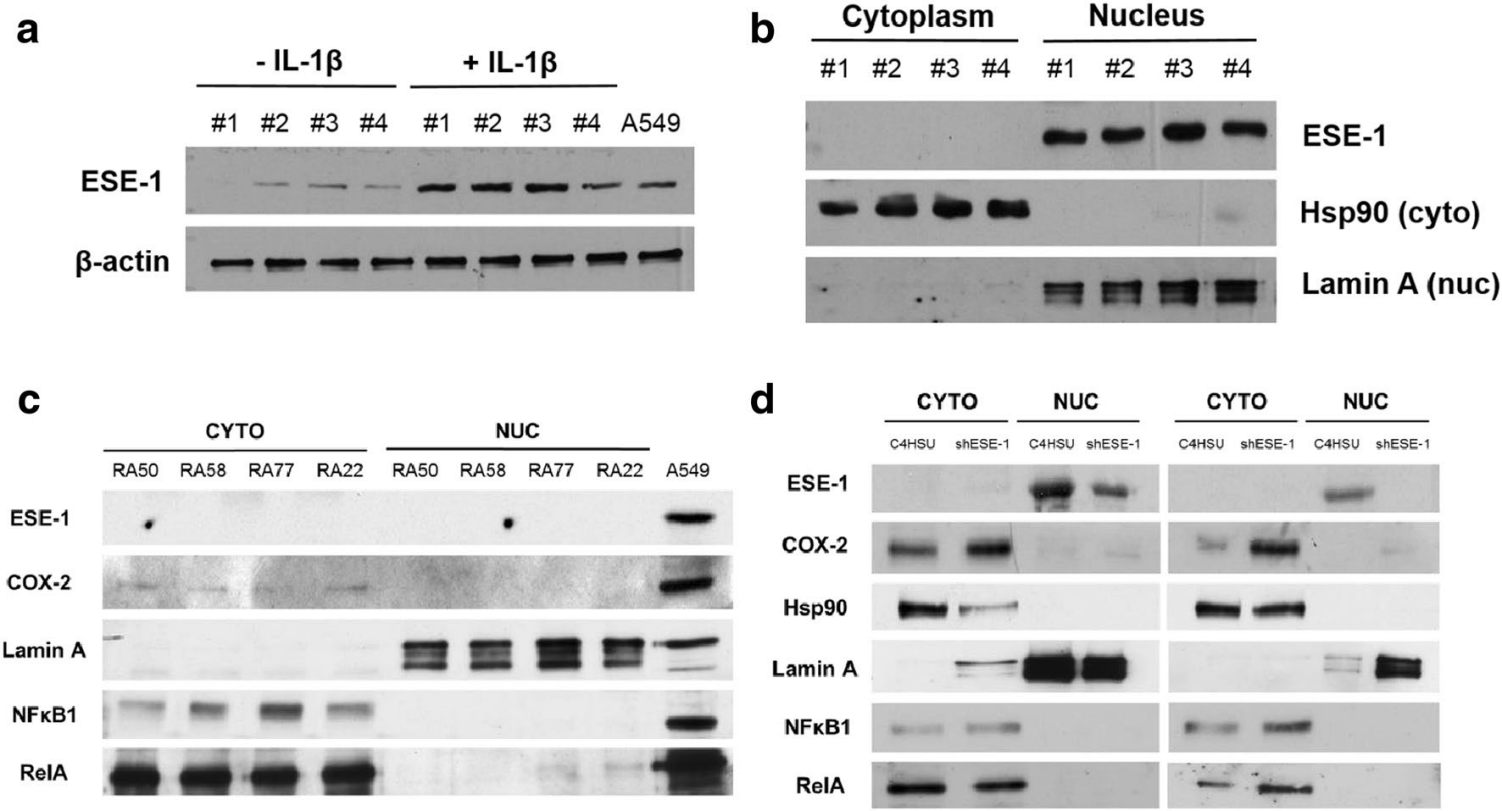
ESE-1 is expressed in the nucleus of RASFs. **a** ESE-1 protein level increases with IL-1β stimulation (n = 4), shown by Western blot on whole cell lysates (WCLs) of stimulated or unstimulated RASFs. #1–#4 denotes patients #50, 58, 77, and 22, and A549 lysate was included as positive control for ESE-1 protein. **b** ESE-1 protein is exclusively expressed in the nucleus, with Hsp90 and Lamin A as cytoplasmic and nuclear markers, respectively. Western blot of nuclear fractionated RASFs stimulated with 10 ng/mL IL-1β for 24 h. **c** ESE-1 and COX-2 are minimally present in RASFs without IL-1β stimulation. **d** Knockdown of ESE-1 by HD-Ad-shESE-1 leads to increase in COX-2 expression in the cytoplasm in IL-1β treatment in RASFs compared to C4HSU control vector. *Western blot* showing results from two different patient RASFs

ESE-1 was previously detected in the cytoplasm by immunostaining of RA patient tissue sections [16]. However, cytoplasmic/nuclear fractionation of activated human RASFs in in vitro following 24 h IL-1β stimulation revealed nuclear presence of ESE-1 (Fig. 3a, b), which was also consistent following shESE-1 HD-Ad viral transduction, where nuclear decrease in ESE-1 led to cytoplasmic increase in COX-2 (Fig. 3d). Furthermore, despite findings of ESE-1 cooperating with NFκB to transactivate target genes such as *iNOS* in endothelial cells [34] and *COX*-*2* in macrophages [17], NFκB was localized in the cytoplasm by 24 h post IL-1β stimulation in RASFs (Fig. 3c, d). This was consistent with the previous finding where NFκB activated by IL-1β in RASFs resolved and returned to normal levels by 4 h of IL-1β stimulation [29]. Therefore while NFκB may be responsible for the transcriptional upregulation of ESE-1 [23], it seems unlikely that NFκB is involved at the 24 h time point when ESE-1 knockdown enhances COX-2 expression. Nuclear localization of ESE-1 is in alignment with its known function as a transcription factor, and it may regulate other genes implicated in COX-2 regulation.

### SW982 cell line shows different pattern of ESE-1 expression from human primary RASFs

**Fig. 4:**
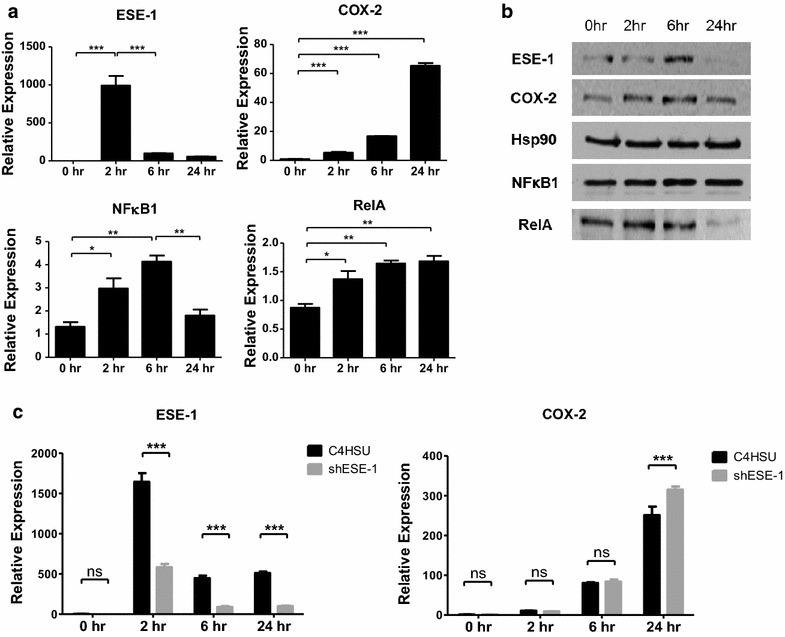
ESE-1 downregulation leads to increased COX-2 mRNA in SW982 cell line. **a** Analysis of ESE-1, COX-2, RelA and NFκB1 transcriptional levels by RT-qPCR in SW982 cells during IL-1β (10 ng/mL) stimulation. **b** Representative Western blot of SW982 stimulated with IL-1β (10 ng/mL) over the 24 h time period. **c** Time course analysis of SW982 cells transduced with C4HSU or shESE-1 HD-Ad vectors and stimulated with IL-1β over 24 h, started 72 h post-transduction. *P < 0.05, **P < 0.01, ***P < 0.001, by one-way ANOVA Bonferroni’s post-test, data shown are representative of two independent experiments with n = 3 each

The transcriptional expression pattern of ESE-1 in response to IL-1β has been studied in numerous non-epithelial cell lines, including human chondrocytes (T/C28a2, C28/I2, and C20A4), osteoblasts (LB-12), monocytes (THP-1), gliomas (U-138 MG and U-373 MG), and endothelial cells (HUVECs), where ESE-1 was shown to be one of the few Ets factors that were specifically responsive to IL-1β-mediated activation, with typical induction pattern of peak expression between 2–6 h and dissipation by 24 h in most cell types [15–17, 34]. Studies have shown that SW982 synovial sarcoma cell line is representative of human primary synovial fibroblasts [35, 36]. However, the expression pattern of ESE-1 in SW982, where ESE-1 peaked at 2 h and underwent drastic downregulation at 24 h (Fig. 4a), and protein expression peaking at 6 h and subsequently undergoing degradation (Fig. 4b), was different from that of RASFs, indicating that it is not a good representative cell line for our purposes. The knockdown of ESE-1 still had a visible effect on COX-2 upregulation at only 24 h (Fig. 4c), when ESE-1 protein was minimally present. p65 (= RelA) also dissipated by 24 h of IL-1β, which made the performance of ChIP very difficult (data not shown). Therefore, it is possible that ESE-1 plays an indirect role or has other unknown function in COX-2 expression, such as mRNA stability, not just acting as a transcription factor, and the function of ESE-1 as a transcript, for example as a competing endogenous RNA, has never been explored.

### ESE-1/Elf3 knockout (KO) macrophages also show increased COX-2 mRNA expression

**Fig. 5:**
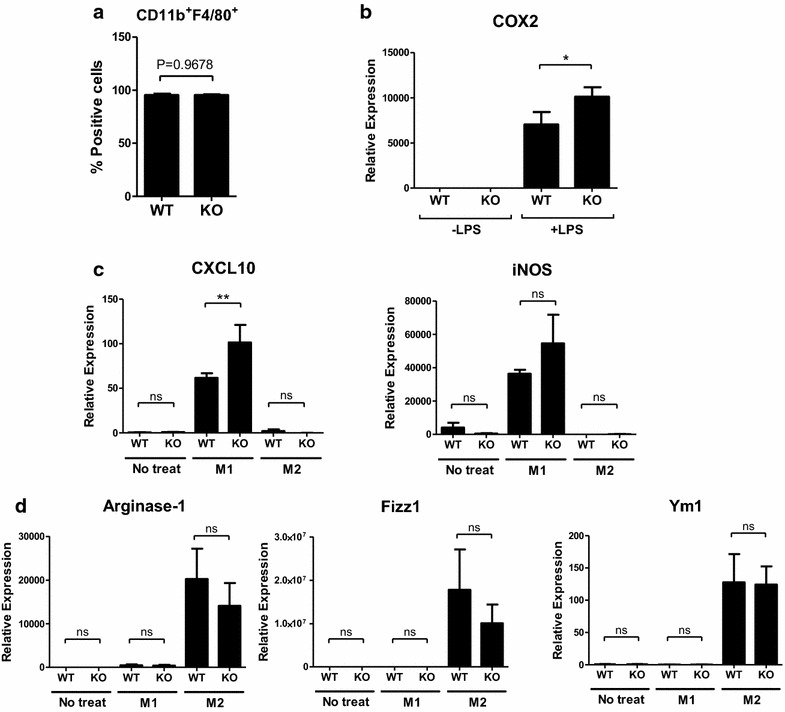
ESE-1 knockout (KO) bone marrow derived macrophages (BMDMs) show increased COX-2 expression and an increased propensity towards M1 phenotype. **a** Expression of macrophage maturation markers CD11b and F4/80 in BMDMs derived from WT or Elf3 KO C57BL/6 mice. Bone marrow cells isolated from the WT or Elf3 mice were differentiated ex vivo in 20 % L-929-conditioned media and analyzed by flow cytometry. The* graph* shows n = 12 for WT and KO mice. **b** ESE-1 knockout BMDMs show increased COX-2 mRNA following LPS (100 ng/mL) treatment. BMDMs were plated at 4 x 10^5^/well on 6-well plates and stimulated with 100 ng/mL LPS or 10 ng/mL IL-4 for 18 h, after which they were lysed for RNA isolation and qPCR analysis (n = 6). **c** shows mRNA expression of genes related to M1 polarization and **d** M2-related genes in WT and KO BMDMs (n = 6) treated with 100 ng/mL LPS (“M1”) and 10 ng/mL IL-4 (“M2) for 18 h prior to analysis. Statistical analysis by one-way ANOVA with Bonferroni’s post-test for selected pairs, *ns* = not significant, *P < 0.05, **P < 0.01

ESE-1 was previously shown to modulate COX-2 in RAW267.4 cells [17]. Therefore, to address the difference in cell type, we also examined COX-2 levels in ESE-1/Elf3 knockout (KO) bone marrow-derived macrophages (BMDMs). Use of Elf3 KO BMDMs circumvented having to expose cells to additional inflammation from transfection- or transduction-mediated gene manipulation. In vitro differentiated BMDMs by CD11b and F4/80 staining were almost 100 % pure (Fig. 5a), and Elf3 KO BMDMs showed no defect in macrophage differentiation which could affect its function [28], expressing normal levels of M-CSFR, Ly-6G, and Ly-6C (data not shown). When subjected to LPS, however, Elf3 KO BMDMs showed increased transcriptional level of COX-2 (Fig. 5b). There are two discrete classes of macrophages, namely M1, which is proinflammatory or classically activated, and M2, which is alternatively activated and takes on more immune-regulatory role. Classically activated M1 macrophages have increased production of pro-inflammatory cytokines such as TNF-α and IL-12, IL-23, nitric oxide (NO), and reactive oxygen species (ROS) and have increased antigen presentation and microbicidal activity, while M2 macrophages typically produce anti-inflammatory cytokines such as IL-10, IL-1 receptor antagonist (IL-1rα) and promote tissue remodeling and repair [37]. Because COX-2 is one of the hallmark genes of M1 [37, 38], we also checked other genes related to M1- versus M2-polariation and observed that Elf3 KO BMDMs were slightly skewed towards M1 (Fig. 5c, d) by polarization-specific gene expression. A single knockout of Elf3 out of almost 30 Ets factors, however, may not show a dramatic effect due to compensation from other Ets, which adds complexity. Elf3 KO BMDMs in fact tended to have more Peas3 and Elf5 and KO lungs expressed higher levels of Erm (data not shown). Nevertheless, it is first time showing ESE-1 can have anti-inflammatory role in macrophages which may be subjected to further analysis.

## Discussion

It is undisputable that understanding the pathogenesis of RA is critical for its prevention and treatment. However, persistent inflammation arises not only from persistent elicitation but also from incomplete resolution, and in pursuit of finding causative mechanisms, primary focus on effectors of pro-inflammatory response may have left some effectors playing dual or complex roles unrecognized from experimental approaches chosen to demonstrate one relationship but not the other. Our study illustrates one such example with an Ets transcription factor, ESE-1. ESE-1 was previously shown to co-operate with NFκB and positively regulate COX-2 by binding to the Ets-binding site on the COX-2 promoter [17]. However, by gene knockdown approach, we made an opposite observation in human RASFs, where knockdown of ESE-1 led to an upregulation of COX-2, which correlated with increased levels of PGE_2_. The use of HD-Ad-mediated knockdown is advantageous over previously employed conventional transfections, given its higher efficacy of gene delivery and long-term expression, as well as much lower cellular toxicity and immunogenicity.

It is important to note that previous findings on ESE-1 have been based on overexpression studies, but with insufficient consideration on the effect of the transfection procedure itself. Ectopic gene expression can lead to supraphysiological levels of the gene of interest, as well as cell toxicity from the transfection. Also, overexpression by transfection may not accurately reflect the temporal behavior of a protein, and may thus lead to artificial interaction or co-localization of proteins that normally do not co-exist under physiological conditions. For example, in RASFs, NFκB is resolved within 4 h following IL-1β stimulation [29], yet the effect of ESE-1 knockdown was only evident at 24 h time point, when the initial inflammation induced by IL-1β had mostly resolved and ESE-1 and NFκB were disparately localized in the nucleus and cytoplasm, respectively. Also, knockdown of ESE-1 had no effect on COX-2 induction in RASF or SW982, indicating that ESE-1 may not play a direct role in regulating COX-2 transcription as previously thought. Rather, given that PGE_2_ has been shown to prolong COX-2 mRNA half-life through the p38 MAPK pathway, ESE-1 may be functioning more as a downstream effector of PGE_2_ signaling than IL-1β at the 24-h time point. In fact, transcriptional activation of COX-2 in NIH 3T3 fibroblasts by PGE_2_ was found to require C/EBP and CRE-1 sites but not NFκB [39], suggesting that ESE-1 may be interacting with protein partners other than NFκB or assuming other functions at later time points.

COX-2 regulation is complex, and occurs at both transcriptional and non-transcriptional levels. The exact transcription factor complexes that are recruited at the COX-2 promoter site vary by cell type and stimulation [40]. Sequence analysis of the 5′-flanking region of the human COX-2 gene has identified several potential transcriptional regulatory elements, including two nuclear factor kappa B (NF-κB) sites, an SP1 site, a CAAT enhancer binding protein (C/EBP), nuclear factor for interleukin-6 expression (NF-IL6) motif, two AP-2 sites, an E-box, and a TATA-box, as well as a peroxisome proliferator response element (PPRE), two cyclic AMP response elements (CRE), and a sterol response element (SRE) [41]. Additionally, COX-2 can be regulated post-transcriptionally by its mRNA stability with the involvement of molecules such as HuR, microRNA 101a and 199a and alternative polyadenylation [42] and by long-noncoding RNAs [43], as well as at its protein level by *N*-glycosylation at Asn-594 and by substrate-dependent suicide inactivation [44]. Therefore, elucidation of the exact role of ESE-1 in COX-2 regulation warrants a separate study.

However, one possible mechanism by which ESE-1 exerts its repressor function on COX-2 transcription may be through ESE-3, another closely related Ets factor and a direct target gene of ESE-1. Unlike ESE-1 which typically peaks at 2 h following cytokine stimulation, ESE-3 was found to peak around 24 h in human airway epithelial cells [23]. While Wu et al. did not consider the possibility of ESE-3 acting as a reciprocal repressor of ESE-1, their data indicates that overexpression of ESE-3 was in fact more effective in inhibiting ESE-1 transcription than ESE-1 itself [23]. Similarly, p38 MAPK plays a crucial role in prolonging COX-2 mRNA stability by PGE_2_ [45, 46], and ESE-3 is known to act as a downstream repressor of p38 MAPK pathway under certain conditions [47]. Therefore, it is possible that ESE-3 acts as a reciprocal repressor of ESE-1 at later time point when IL-1β is degraded, and that this feedback loop is defective in RASFs. Given that ESE-3 is also upregulated during stress-induced senescence in human fibroblasts [48], dysregulation in the ESE-1-ESE-3-MAPK regulatory loop may be involved in sustaining the non-senescent phenotype of RASFs.

Lastly, although knockdown of ESE-1 also resulted in upregulation of COX-2 in SW982 cells, the pattern of ESE-1 expression induced by IL-1β was very different, indicating SW982 is not a truly representative model to study the role ESE-1 in human RASFs in vitro. Because most of the ESE-1 targets have been identified in immortalized cell lines, this points to the need to develop better cell model systems that more closely mimic RASFs in situ, as well as experimental tools which minimally interfere with immune-responsive function of the target proteins. Nonetheless, our findings reveal new insights into the role of ESE-1 in rheumatoid arthritis, as it is the first time to demonstrate that ESE-1 may assume an anti-inflammatory role under physiological conditions to prevent excessive tissue damage during an inflammatory response, by negatively regulating COX-2 in human RASFs.

## Conclusions

ESE-1 acts as a negative regulator of COX-2 in human RASFs and its effect on COX-2 is NFκB-independent, occurring at later phases of an inflammatory response. This may indicate that ESE-1 is involved in the resolution of inflammation unlike previously thought, and this discrepancy may be attributed to confounding responses to transfection procedures.

## Additional files

10.1186/s13578-016-0105-7 RT-qPCR primer sequences used in the study.
10.1186/s13578-016-0105-7 Additional patient data for Figure 1 and Figure 2.
10.1186/s13578-016-0105-7 qPCR analysis of ESE-1 knockdown in human RASFs (n = 6) for (A) MMP-1 and MMP-13, and (B) NFκB1 and RelA. ns = not significant.

## Abbreviations

AA: arachidonic acid
AP-1: activator protein 1
BMDM: bone marrow-derived macrophage
C4HSU: control helper-dependent adenoviral vector
cDNA: complementary deoxyribonucleic acid
C/EBP: CAAT enhancer binding protein
COX-2: cyclooxygenase-2
CRE: cyclic AMP response elements
DEAE-Dextran: diethylaminoethyl-dextran
DMEM: Dulbecco’s Modified Eagle Medium
Elf3: E74-like factor 3
ELISA: enzyme-linked immunosorbent assay
ESE-1: epithelium-specific Ets transcription factor-1
Ets: E26 transformation-specific domain
FBS: fetal bovine serum
GAPDH: glyceraldehyde-3-phosphate dehydrogenase
HD-Ad: helper-dependent adenovirus
Hsp90: heat shock protein 90
IL-1β: interleukin-1β
KO: knockout
LPS: lipopolysaccharide
MAPK: mitogen-activated protein kinase
MMP: matrix metalloproteinase
MOI: multiplicity of infection
mRNA: messenger RNA
NF-IL6: nuclear factor for interleukin-6 expression motif
NFκB: nuclear factor kappa B
NO: nitric oxide
NSAIDs: non-steroidal anti-inflammatory drugs
PG: prostaglandin
PGE_2_: prostaglandin E_2_
PGH_2_: prostaglandin H_2_
PPRE: peroxisome proliferator response element
RA: rheumatoid arthritis
RASF: rheumatoid arthritis synovial fibroblast
RNA: ribonucleic acid
shRNA: short-hairpin RNA
ROS: reactive oxygen species
RPMI: Roswell Park Memorial Institute medium
RT-qPCR: quantitative reverse transcription polymerase chain reaction
SRE: sterol response element
TCP: Toronto Centre for Phenogenomics
TNF-α: tumor necrosis factor-α
WT: wild-type
