# Supplementary material for: Epithelium-specific Ets transcription factor-1 acts as a negative regulator of cyclooxygenase-2 in human rheumatoid arthritis synovial fibroblasts
Source: Cell Biosci. 2016 Jun 16;6:43. doi: 10.1186/s13578-016-0105-7 (PMC4910355; doi:10.1186/s13578-016-0105-7)
Supplement: Supplementary file 1 — 10.1186/s13578-016-0105-7 RT-qPCR primer sequences used in the study. [file 13578_2016_105_MOESM1_ESM.pdf]

Supplementary Table 1. RT-qPCR primer sequences used in the study

Gene name	Primer Sequence	Source/Reference
**Human**		
ESE-1	5’-GGCGTCTTCAAGTTCCTGCG-3’ (Forward)<br>5’-CTCCCGTTTGTAGTAGTACCT-3’ (Reverse)	[22]
COX-2	5’-CTGGCGCTCAGCCATACAG-3’ (Forward)<br>5’-CGCACTTATACTGGTCAAATCCC-3’ (Reverse)	Harvard Primer Bank ID223941909c1
NFkB1	5’-GAGGATGGGATCTGCACTGT-3’ (Forward)<br>5’-TTCGTGCTTCCAGTGTTTCA-3’ (Reverse)	[49]
RELA	5’-ACTTCCCCTGCTCTCCTGAT-3’ (Forward)<br>5’-CAACCCCCTTCCTCCTATTC-3’ (Reverse)	[49]
MMP-1	5’-GGGGCTTTGATGTACCCTAGC-3’ (Forward)<br>5’-TGTCACACGCTTTTGGGGTTT-3’ (Reverse)	Harvard Primer Bank ID225543092c3
MMP-13	5’-CTGGCATGACGCGAACAAA-3’ (Forward)<br>5’-TCCCAGGAATTGGTGATAAGTAGA-3’ (Reverse)	Invitrogen Cat# 10336022
GAPDH	5’-GAAGGTGAAGGTCGGAGTC-3’ (Forward)<br>5’-GAAGATGGTGATGGGATTTC-3’ (Reverse)	[22]
**Mouse**		
COX-2	5’-AGCCAGGCAGCAAATCCTT-3’ (Forward)<br>5’-ATTCCCCACGGTTTTGACA-3’ (Reverse)	[50]
CXCL-10	5’-TGAATCCGGAATCTAAGACCATCAA-3’ (Forward)<br>5’-AGGACTAGCCATCCACTGGGTAAAG-3’ (Reverse)	[51]
iNOS	5’-CACGCTTGGGTCTTGTTCACT-3’ (Forward)<br>5’-TGGGTCCTCTGGTCAAACTCTT-3’ (Reverse)	[52]
Arginase-1	5’-GCTGTCTTCCCAAGAGTTGGG-3’ (Forward)<br>5’-ATGGAAGAGACCTTCAGCTAC-3’ (Reverse)	[53]
Fizz1	5’-TCCCAGTGAATACTGATGAGA-3’ (Forward)<br>5’-CCACTCTGGATCTCCCAAGA-3’ (Reverse)	[54]
Ym1	5’-GGGCATACCTTTATCCTGAG-3’ (Forward)<br>5’-CCACTGAAGTCATCCATGTC-3’ (Reverse)	[54]
GAPDH	5’-GTGGCAAAGTGGAGATTGTTGCC-3’ (Forward)<br>5’-GATGATGACCCGTTTGGCTCC-3’ (Reverse)	[22]
