# Supplementary figures and images for: Epithelium-specific Ets transcription factor-1 acts as a negative regulator of cyclooxygenase-2 in human rheumatoid arthritis synovial fibroblasts
Source: Cell Biosci. 2016 Jun 16;6:43. doi: 10.1186/s13578-016-0105-7 (PMC4910355; doi:10.1186/s13578-016-0105-7)

# Supplementary Figure 1

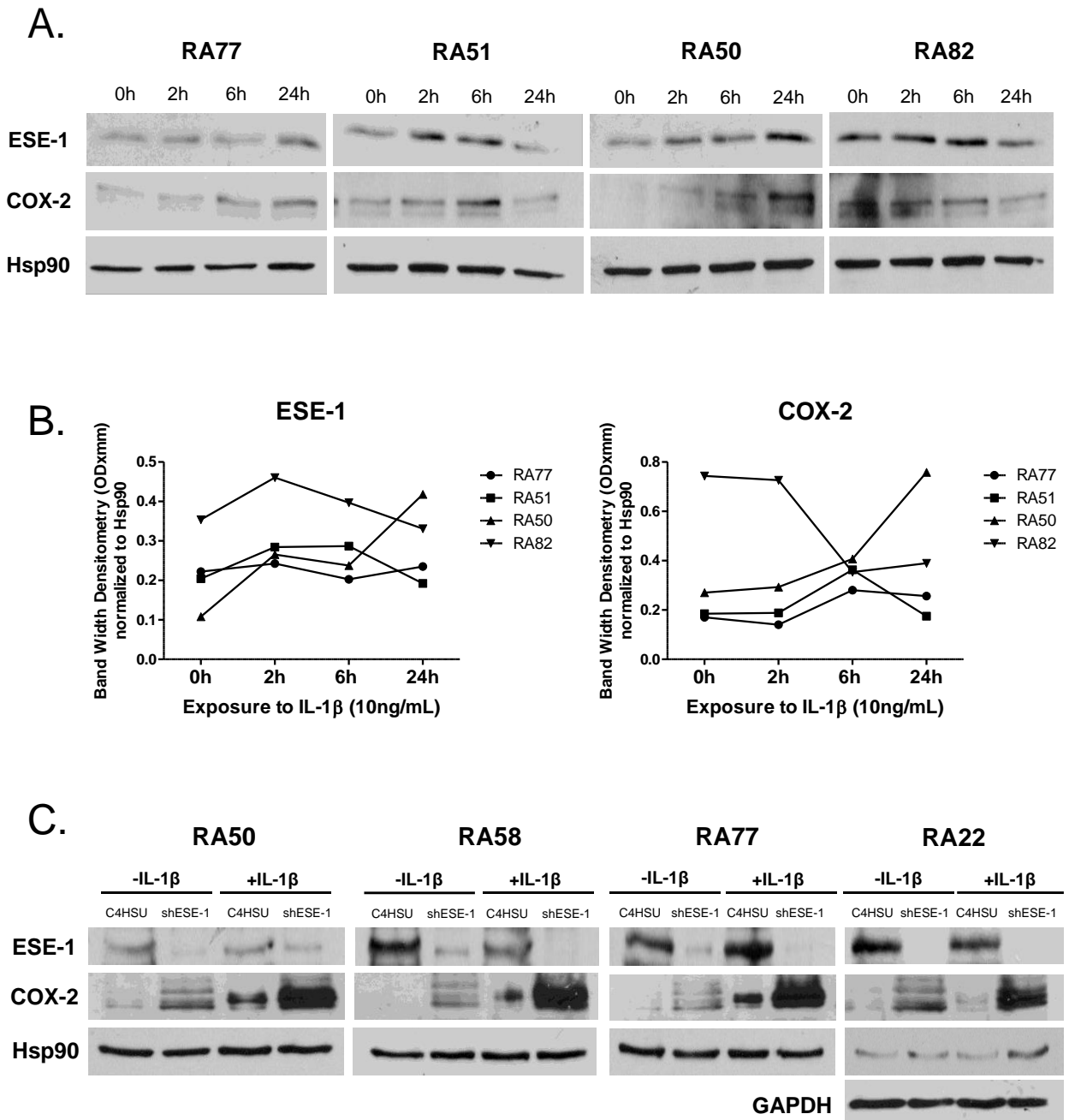

Supplement: Supplementary file 2 — 10.1186/s13578-016-0105-7 Additional patient data for Figure 1 and Figure 2. [file 13578_2016_105_MOESM2_ESM.pdf]

## Supplementary Figure 2

A.

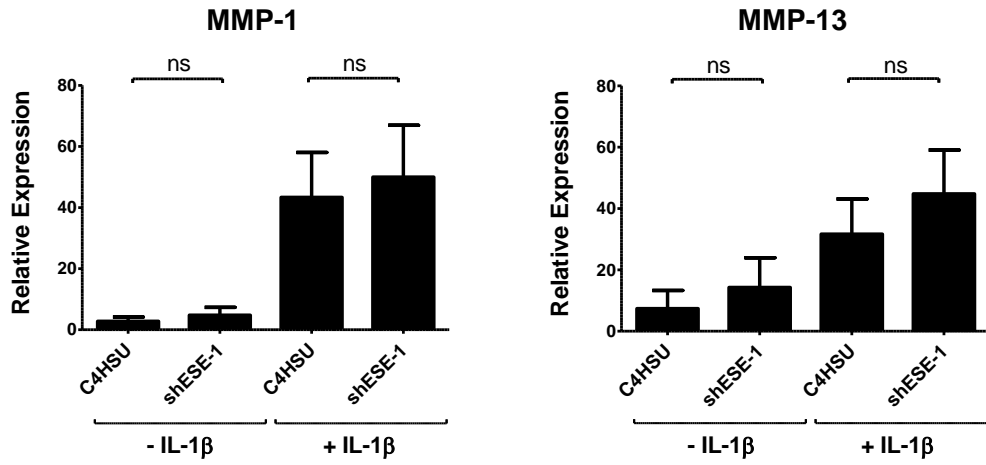

B.

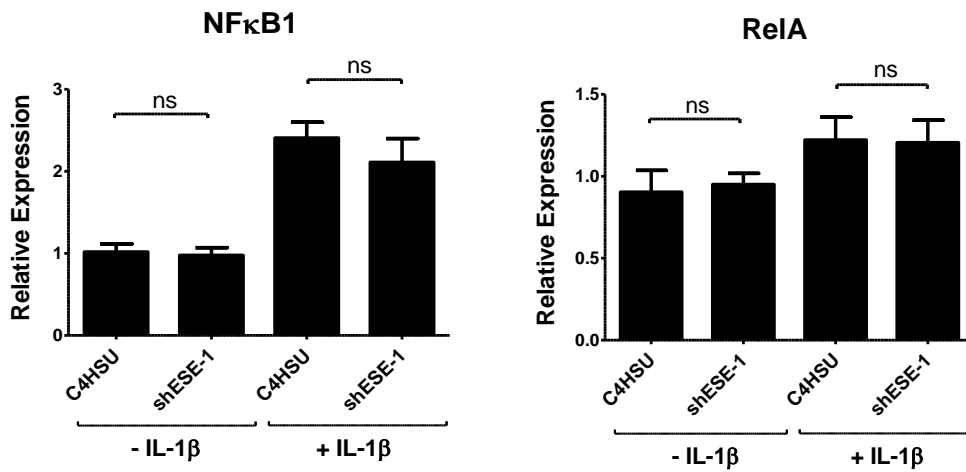

Supplement: Supplementary file 3 — 10.1186/s13578-016-0105-7 qPCR analysis of ESE-1 knockdown in human RASFs (n = 6) for (A) MMP-1 and MMP-13, and (B) NFκB1 and RelA. ns = not significant. [file 13578_2016_105_MOESM3_ESM.pdf]
